# Experimental and Modeling Study of the Nanofiltration of Alcohol-Based Molecules and Amino Acids by Commercial Membranes

**DOI:** 10.3390/membranes13070631

**Published:** 2023-06-29

**Authors:** Shirin Shahgodari, Jordi Labanda, Joan Llorens

**Affiliations:** Department of Chemical Engineering and Analytical Chemistry, University of Barcelona, Martí i Franquès 1, 08028 Barcelona, Spain; shirin.shahgodari@ub.edu (S.S.); jllorensl@ub.edu (J.L.)

**Keywords:** nanofiltration membranes, transport model, membrane pore radius, concentration polarization, amino acid rejection

## Abstract

The nanofiltration performance of three commercial membranes was analyzed by the Steric Pore Model (SPM) and the extended Nernst–Planck diffusion equation inside membrane pores. The model was completed with the equation to predict the concentration polarization, and the mass transfer coefficient was determined by considering the presence of a feed spacer. The model parameters that characterized the performance of the membrane were the hydrodynamic coefficient, which accounts for the possible variations in solute size and membrane pore radius, the effective membrane thickness, and the water permeability coefficient. All experiments were conducted at fixed feed pH of 6. The rejections of uncharged solutes (glucose for membranes with a high molecular weight cut-off (MWCO) and glycerol and ethylene glycol for membranes with a low MWCO) allowed the model parameters to be determined. We found that glycerol and ethylene glycol overestimate the membrane pore radius due to their ability to interact with the membrane matrix. Therefore, the rejection of glycine as a small amino acid was explored to characterize the membranes with low MWCO since these molecules do not interact with the membrane matrix and have an almost zero charge at pH values between 4.5 and 6.5. Based on the experimental rejections, it was stated that glucose and glycine could be separated by these membranes operating in continuous diafiltration mode.

## 1. Introduction

Initially, nanofiltration (NF) membranes were intensively used to retain monovalent and multivalent inorganic salts, being applied in the water softening of wastewater and even seawater [1]. Since then, their applications have been extended to the rejection of organic solutes in industrial and municipal waters [2,3]. One promising application of membranes is in food processing [4], for example, in the sugar industries [5].

NF membranes contain three porous stacked layers of different polymers. The top layer (also called the active layer) is composed of an imperfect crosslinking polymer containing ionizable groups, which confer a specific charge to the membrane depending on the characteristics of the feed solutions [6]. In general, NF membranes are amphoteric, positively charged, negatively charged, or have zero net charge, depending on the pH of the feed solution [7,8]. Because of this variation, each membrane exhibits a characteristic isoelectric point (IEP) [8]. In addition, the effective charge density of the membrane depends on the composition of the feed solution due to the adsorption of cations and counterions onto the membrane surface [9,10].

Nanofiltration performance can be described by the Spiegler–Kedem Model, which is based on irreversible thermodynamics [11] and determines the solvent and solute fluxes directly from the difference in the chemical potential between the two sides of the membrane. It does not include, in a direct form, either the interaction between the solvent and the membrane or the process of diffusion within the membrane. Nanofiltration can also be described by mechanistic models [12], based on the partitioning equilibrium at the solution–membrane interface and on the solute transport through the membrane pores, which is generally described by the extended Nernst–Planck equation [12]. The ion flux through the membrane depends on the convention flux, ion diffusivity, and the potential gradient inside the pores [13].

The basic Steric Pore Model (SPM) considered only steric hindrance in the partitioning at the solution–membrane interface, which is the overall mechanism for the membrane process where the applied pressure is the driving force [13]. Solutes with a molecular size larger than the membrane pore radius cannot permeate the membrane. Hence, the steric exclusion depends on the ratio of the solute to the pore radius, which becomes relevant as the solute radius approaches the pore radius [14]. The Donnan effect describes the electrostatic interactions between charged solutes and the superficial membrane, which generates a membrane potential that can hinder the solutes [15]. This model is called the Donnan–Steric Pore Model (DSPM). To solve the DSPM, a constant Donnan potential across the membrane is assumed and the effective membrane charge density can be calculated by fitting the experimental rejections [12]. Some studies have proposed the estimation of membrane charge using adsorption isotherms to quantify the adsorption of ions onto a membrane surface [16]. These approximations can help to understand the mechanism of membrane charge formation in solutions with varying ion concentrations and pH [17,18].

The DSPM can be improved by considering dielectric exclusion, which can arise from the difference in the dielectric constant of the bulk solution between the membrane matrix and/or inside the pores [19]. Thus, two different mechanisms in dielectric exclusion can be considered: image forces and ion solvation [20], also called the Born equation. In both cases, the exclusion depends on the square of the ion charge. The image force interaction is also affected by the Donnan potential. The fixed charges on the membrane surface induce an interaction between the ions in the solution and the polarized charges (also called image forces) at the solution–membrane interface [19,21]. Dielectric exclusion based on ion solvation involves a reorientation of a single layer of water molecules within the pore walls, which increases the solvation energy of the ions and reduces viscosity and the dielectric constant inside the pores compared to that of the bulk solution [22,23].

The aim of the present study was to evaluate the performance of three commercial nanofiltration membranes with different uncharged and zero-charged organic solutes using the Spiegler–Kedem model in combination with film-theory quantifying the concentration polarization, the SPM as partitioning equilibrium and the extended Nernst–Planck equation. First, the nanofiltration of uncharged solutes (glucose, glycerol, and ethylene glycol) was carried out to determine the effective membrane pore radius, which may depend on the properties of the molecules since glycerol and ethylene glycol are organic solvents that can modify the membrane matrix and lead to an overestimation of the membrane pore radius. The estimated value was validated with zero-charged organic molecules, such as amino acids, since their rejections are not affected by the electrostatic interactions with the membrane surface at pH 6.

## 2. Theory

The permeate flux, $J_{v}$, is defined as the product of the water permeability coefficient, $L_{p}$, and the driving force, which is calculated by the difference in effective transmembrane pressure, $\Delta P_{e}$.

(1) $$J_{v}=L_{p}\;\Delta P_{e}$$

The most common expression is derived from the principle of irreversible thermodynamics in reverse osmosis membranes with low solute rejections and, therefore, it is assumed to be valid for NF membranes [11]. The same expression can be obtained by alternative models, such as the well-known Hagen–Poiseville equation. In the presence of concentration polarization, the concentration on the feed/membrane surface is higher than the feed bulk solution, and the general equation for the permeate flux for multicomponent solutions is expressed as:

(2) $$J_{v}=L_{p}\cdot (\Delta P-\sum_{s=1}^{n}\sigma_{v,s}\;\Delta \pi_{s}^{w})$$

where ΔP is the applied transmembrane pressure, $\sigma_{v,s}$ is the Staverman reflection coefficient of the solute *s*, $\Delta \pi_{s}^{w}$ is the is the transmembrane osmotic pressure difference at the feed/membrane interface for the solute *s*, and *n* is the number of solutes in the solution. Staverman reflection coefficient can be calculated by considering the osmotic pressure differences in the feed/membrane and membrane/permeate interfaces as [24]:

(3) $$\sigma_{v,s}=1-\frac{\Delta \pi_{s}^{m}}{\Delta \pi_{s}^{w}}$$

where $\Delta \pi_{s}^{m}$ is the is the transmembrane osmotic pressure difference in the feed/membrane interface at the membrane side for the solute *s*, which is calculated by the difference between the osmotic pressure inside the membrane at the feed face and at the permeate face. These osmotic pressure differences can be calculated by the solute concentrations at the feed/membrane interface from the solute concentration in the bulk solution.

According to the film theory model, the relationship between the feed bulk concentration and the concentration at the feed/membrane interface is given by the mass transfer coefficient from the concentration polarization layer to the feed solution, $k_{s}$. The permeate flux is also calculated as follows:

(4) $$J_{v}=k_{s}\;ln\left(\frac{c_{1,s}^{w}-c_{p,s}}{c_{1,s}-c_{p,s}}\right)$$

where $c_{1,s}^{w}$ is the solute concentration at the feed/membrane interface, $c_{1,s}$ is the solute concentration in the bulk solution, and $c_{p,s}$ is the solute concentration at the membrane/permeate interface, which is considered to be equal to solute permeate concentration.

Mass transfer coefficients may be determined using the Sherwood relationship, which depends on the Reynold, *Re*, and Schmidt, *Sc*, numbers:

(5) $$Sh=\frac{k_{s}\;d_{h}}{D_{s}}=a\;Re^{b}\;Sc^{c}$$

where $d_{h}$ is the hydraulic diameter of the feed chamber, $D_{s}$ is the solute diffusion coefficient, and *a*, *b*, and *c* are empirical coefficients that depend on hydrodynamic conditions and NF cell geometry. The Reynolds number is calculated as $Re=u\;d_{h}/\nu$ and the Schmidt number as $Sc=\upsilon /D_{s}$, where *u* is the main crossflow velocity, and *ν* is the kinematic viscosity.

On the other hand, the solute concentration in the feed/membrane interface at the membrane side may be estimated by the membrane equilibrium partitioning expression. Based on the SPM, the general equation of the equilibrium partitioning for uncharged solutes is defined only by the steric exclusion mechanism as:

(6) $$\frac{\gamma_{1,s}^{m}\;c_{1,s}^{m}}{\gamma_{1,s}^{w}\;c_{1,s}^{w}}=\emptyset_{s}={\left(1-\lambda_{s}\right)}^{2}$$

where $c_{1,s}^{m}$ is the solute concentration at the feed/membrane interface on the membrane side, $\gamma_{1,s}^{w}$ and $\gamma_{1,s}^{m}$ are the activity coefficients at the feed/membrane interface on the solution side and on the membrane side, respectively, $\emptyset_{s}$ is the steric partitioning coefficient of the solute *s*, which may be calculated considering cylindrical pore geometry, and $\lambda_{s}$ is the hydrodynamic coefficient of the solute *s* that is defined as the ratio between the solute radius and mean membrane pore radius ($\lambda_{s}=r_{s}/r_{p}$). For solutes with similar size of membrane pore radius, the hydrodynamic coefficient tends to unity, the steric partitioning coefficient tends to zero, and the solute concentration on the membrane side tends to zero, and the solute is totally rejected. A similar partitioning equation can be written for the membrane/permeate interface to calculate the solute concentration in the permeate stream from the concentration on the membrane side, $c_{2,s}^{m}$:

(7) $$\frac{\gamma_{2,s}^{m}\;c_{2,s}^{m}}{\gamma_{p,s}\;c_{p,s}}=\emptyset_{s}={\left(1-\lambda_{s}\right)}^{2}$$

The equilibrium partitioning equations allow calculation of the Staverman reflection coefficient with a very simple equation that depends only on the hydrodynamic coefficient if the Van’t Hoff equation for the estimation of osmotic pressure is assumed:

(8) $$\sigma_{v,s}=1-\frac{\emptyset_{s}\;\Delta \pi_{s}^{w}}{\Delta \pi_{s}^{w}}=1-\emptyset_{s}$$

Membrane effectiveness in the separation of components is usually estimated by the calculation of the observed and intrinsic solute rejections:

(9) $$R_{obs,s}=1-\frac{c_{p,s}}{c_{1,s}}$$

(10) $$R_{int,s}=1-\frac{c_{p,s}}{c_{1,s}^{w}}$$

The observed rejection is easily calculated from the experimental data, with $c_{1,s}$ being as the feed solute concentration. Nevertheless, the intrinsic rejection must be calculated with equations, since $c_{1,s}^{w}$ cannot be measured experimentally. An expression for intrinsic rejection may be obtained by combining the solute flux across the membrane, $J_{s}$, and the partitioning equilibrium. The solute transport across the NF membranes is described by the extended Nernst–Planck equation, which considers the solute diffusion, due to the concentration gradient, and the solute convention inside the membrane pores [25]. This equation assumes no interaction between solute fluxes and can be written for uncharged solutes by considering an ideal solution that is independent of the pressure gradient, as follows:

(11) $$J_{s}=C_{p,s}\;J_{v}=-D_{s,p}\left(\frac{\varepsilon }{\tau }\right)\;\frac{dC_{m,s}}{dx}+K_{c,s}\;C_{m,s}\;J_{v}$$

where $C_{m,s}$ is the solute concentration across the membrane pores, ε/τ is the porosity–tortuosity ratio, $D_{s,p}$ is the solute diffusion coefficient inside the membrane pores, and $K_{c,s}$ is the convective hindrance factor, which represents the effects of the pore walls on the convection movement. $D_{s,p}$ is calculated from the solute diffusion coefficient in bulk solution, $D_{s}$, by the diffusive hindrance factor ($D_{s,p}=K_{d,s}\;D_{s}$). The two hindrance factors depend on the hydrodynamic coefficient and can be calculated following the cylindrical pore geometry [26]:

(12) $$K_{c,s}=\frac{1+3.867\;\lambda_{s}-1.907\;\lambda_{s}^{2}-0.834\;\lambda_{s}^{3}}{1+1.867\;\lambda_{s}-0.741\;\lambda_{s}^{2}}$$

(13) $$K_{d,s}=\frac{1}{\emptyset_{s}}\;\left(1+\frac{9}{8}\;\lambda_{s}\ln\left(\lambda_{s}\right)-1.56034\;\lambda_{s}+0.528155\;\lambda_{s}^{2}+1.91521\;\lambda_{s}^{3}-2.81903\;\lambda_{s}^{4}+0.270788\;\lambda_{s}^{5}+1.10115\;\lambda_{s}^{6}-0.435933\;\lambda_{s}^{7}\right)$$

The integration of Equation (11), using the initial condition ($C_{m,s}=\emptyset_{s}\;c_{1,s}^{w}$ at x=0) and the boundary condition ($C_{m,s}=\emptyset_{s}\;c_{p,s}$ at x=δ) and assuming that the activity coefficients are very close to unity, leads to a simple relationship for the uncharged solute intrinsic rejection:

(14) $$R_{int,s}=1-\frac{K_{c,s}\;\emptyset_{s}}{1-\left(1-K_{c,s}\;\emptyset_{s}\right)\;exp\left(-\frac{K_{c,s}\;\delta }{D_{s,p}}\;J_{v}\right)}$$

where δ is the effective membrane thickness that takes into account the porosity–tortuosity ratio; therefore, the solute intrinsic rejection depends on the membrane pore radius, effective membrane thickness, and volumetric permeate flux. For a given solute and membrane, the solute intrinsic rejection increases with the permeate flux reaching an asymptotic value (maximum and constant value) at very high permeate fluxes ($R_{int,s}=1-K_{c,s}\;\emptyset_{s}$ at $J_{v}\to \infty$).

## 3. Materials and Methods

### 3.1. Experimental Equipment

The experimental device consisted of a reservoir tank, a pump, a closed-pipe pressure dampener to prevent pressure oscillations, pressure gauges, a filtration cell, and flow meters for the retentate and permeate. The three membranes used in this study were organic thin-film composite membranes: SelRO^®^ MPF-36 (Koch, Wilmington, MA, USA), SelRO^®^ MPF-34 (Koch, Wilmington, MA, USA), and Desal-5-DK (GEOsmonics, Le Mee sur Seine, France). According to the manufacturers, the MPF-36 membrane has a nominal MWCO of 1000 g mol^−1^, while both the MPF-34 and DK membranes have an MWCO of approximately 200 g mol^−1^. The MPF-36 and MPF-34 membranes are proprietary thin-film composite membranes, with a maximum operating temperature of 70 °C and a functional pH range of 1–13. While the Desal 5DK membrane is a thin-film composite membrane with a polyamide top layer, and it can operate up to 90 °C, but the optimal pH range is 2–11.

The membranes were placed in a laboratory-scale SEPA CF II membrane element cell (Osmonics, Minnetonka, MN, USA). The feed chamber contained a feed spacer and stainless-steel shims that allowed the reduction of the depth of the chamber—increasing the crossflow velocity. The spacer consists of woven cylindrical filaments in a diamond configuration with an angle between the crossing filaments of 90°. Thus, the feed channel has a hydraulic diameter of 0.50 mm and a porosity of 0.92 [27]. The effective membrane filtration area was 140 cm^2^. The feed flow was introduced into the membrane cell by means of a positive displacement pump equipped with a closed-pipe pressure dampener to prevent pressure oscillations, supplied by CAT-PUMPS (Kontich, Belgium), and the flow circulated tangentially to the membrane and guaranteed a crossflow velocity of 1.5 m s^−1^.

First, the membranes were conditioned with demineralized water at room temperature for 24 h. Then, they were placed in the cell and pressurized with pure water at a constant temperature for 2 h. All experiments were conducted in the steady-state mode, in which both the concentrate and permeate streams were recycled to the feed vessel at a fixed temperature of 20 ± 0.5 °C, with the feed tank immersed in a thermostatic bath. It was previously confirmed that the steady state is reached after 1 h of nanofiltration at a constant pressure. At this point, the permeate flow was measured, and samples of the concentrate and permeate streams were taken to determine the solute concentrations and calculate the observed rejection. Several pieces of the three membranes were used to conduct the experiments, and the observed rejections are the mean value of at least three measurements. The results proved to be highly reproducible, with the standard deviations being lower than 10% of the average rejections.

### 3.2. Feed Solutions and Analytical Techniques

All the chemicals used (glucose, glycerol, ethylene glycol, triglycine, glycine, and sodium chloride) were supplied by Sigma-Aldrich (Madrid, Spain) and were obtained in a pure grade. The properties are shown in Table 1. The pure water used in this study was prepared using a Milli-Q water purification system and filtered through a membrane with a pore diameter of 45 µm. Solute concentrations of alcohol-based molecules were 3.5 and 5 g L^−1^ and amino acid solutions were 1.5 g L^−1^. All solutions were prepared and analyzed at a constant pH of 6, which was adjusted by adding small amounts of HCl or NaOH as needed.

The nanofiltration performance was determined by measuring the solute concentration, conductivity, and pH of the concentrate and permeate streams, as well as the transmembrane pressure and the permeate flow rate. The organic solute concentration was determined as the total organic carbon (TOC) amount using a Shimadzu ASI-V analyzer, while the NaCl concentration and the pH of the solutions were determined from conductivity measurements using the IntelliCAL™ CDC and PHC probes connected to an HQ40d multimeter (Hach, Loveland, CO, USA), respectively.

### 3.3. Modeling Procedure

Membrane performance for the solutions was evaluated by determining the experimental permeate flux, $J_{v}^{exp}$, as the ratio of volumetric permeate flow to membrane area, and the experimental observed solute rejection, $R_{obs,s}^{exp}$, based on the solute concentration measurements in the feed and permeate streams. Nevertheless, the first step to fit the experimental data was to estimate the pure water permeability coefficient, $L_{pw}$, by the measured pure water flux at different transmembrane pressures.

The modeling procedure is based on fitting the experimental data, consisting of the observed rejections as a function of the permeate flux and the permeate flux as a function of transmembrane pressure, in order to obtain the model parameters: hydrodynamic coefficient and the effective membrane thickness. The mass transfer coefficient, which quantifies the concentration polarization, can be calculated from the Sherwood relationship. For a rectangular feed chamber with a commercially available spacer, the mass transfer coefficient can be calculated according to Equation (5) as [28]:

(15) $$k_{s}=0.2\;Re^{0.57}\;Sc^{0.40}\;\frac{D_{s}}{d_{h}}$$

The calculated mass transfer coefficients for the solutes showed relatively high values, ranging from 2.15 × 10^−4^ to 3.05 × 10^−4^ m/s, evidencing a small concentration polarization phenomenon. When the permeate flux is very high (>10 μm·s^−1^), the mass transfer coefficient is corrected with the following correlation [29]:

(16) $$k_{s,hp}=\left[F+{\left(1+0.26\;F^{1.4}\right)}^{-1.7}\right]\;k_{s}$$

where $k_{s,hp}$ is the mass transfer coefficient at high permeate flux and *F* is defined as $F=J_{v}/k_{s}$.

The detailed modeling procedure involves the following steps:

1. Guess the initial values of $\lambda_{s}$ and δ, and calculate the hindrance coefficients according to Equations (12) and (13).
2. Calculate the solute intrinsic rejection as a function of experimental permeate flux with Equation (14) and the observed rejection with the experimental permeate flux and the mass transfer coefficient with the following expression:
  (17) $$R_{obs,s}=\frac{R_{int,s}}{R_{int,s}+\left(1-R_{int,s}\right)\;exp\left(\frac{J_{v}}{k_{s,hp}}\right)}$$
3. Determine the values of *l_s_* and *d* that provided the optimal fit for the experimental solute rejections by minimizing the least-squares objective function, $LS_{R,s}$:
  (18) $$LS_{R,s}=\frac{1}{\sqrt{m-1}}\;\sqrt{\sum_{k=1}^{m}{\left(R_{obs,s,k}^{calc}-R_{obs,s,k}^{exp}\right)}^{2}}$$
  where *m* is the number of transmembrane pressures tested for any of the feed solutions.
4. Guess the solution permeability coefficient value $L_{p}$ to calculate the permeate flux. By combining Equations (2) and (4), the permeate flux can be estimated from the calculated observed solute rejection and applied transmembrane pressure with the following expressions:
  (19) $$Z_{v}\;exp\left(Z_{v}\right)=\frac{\sum_{s=1}^{n}\sigma_{v,s}\;\Delta \pi_{s}\;\exp\left(\frac{L_{p}\;\Delta P}{k_{s,hp}}\right)}{k_{shp}}$$
  (20) $$J_{v}^{calc}=L_{p}\;\Delta P-k_{shp}\;Z_{v}$$

Equation (19) is a Lambert function and can be solved iteratively to find $Z_{v}$.

5. Determine the value of $L_{p}$ that provided the optimal fit for the experimental permeate flux by minimizing the least-squares objective function $LS_{Jv,s}$, between experimental and calculated permeate fluxes at each transmembrane pressure:
  (21) $$LS_{Jv,s}=\frac{1}{\sqrt{m-1}}\;\sqrt{\sum_{k=1}^{m}{\left(J_{v,k}^{calc}-J_{v,k}^{exp}\right)}^{2}}$$

## 4. Results and Discussion

### 4.1. Pure Water Permeate Flux

**Figure 1:**
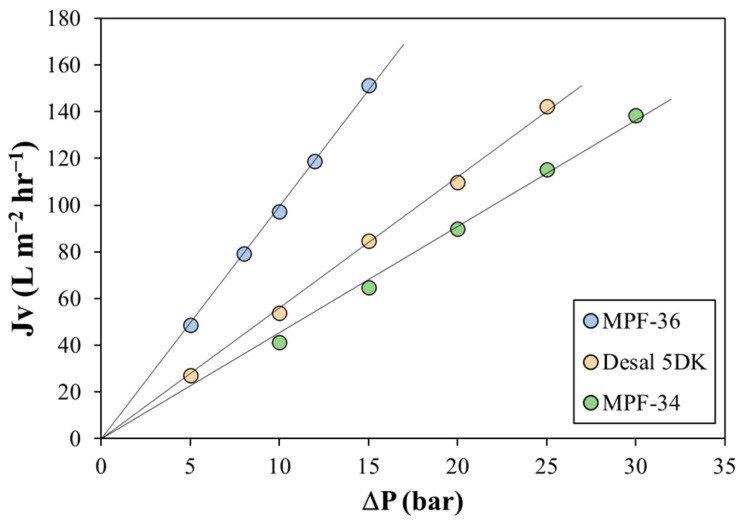
Pure water permeate flux as a function of applied transmembrane pressure for the three membranes. Symbols correspond to the experimental data, and lines correspond to the best linear regression.

First, the pure water permeability coefficient of the three membranes was determined from the slope of the linear relationship between the measured water flux and the transmembrane pressure (Figure 1). A strong linear correlation was observed for the three membranes, with a correlation coefficient (*R*^2^) greater than 0.995. The MPF-36 membrane showed the highest permeability coefficient (9.94 L m^−2^ hr^−1^ bar^−1^), corresponding to the membrane with the highest MWCO. The other two membranes showed a similar pure water permeability coefficient, which was 4.53 L m^−2^ hr^−1^ bar^−1^ for the MPF-34 membrane and 5.58 L m^−2^ hr^−1^ bar^−1^ for the DK membrane. This suggests that the MPF-34 membrane exhibited the greatest resistance to water permeation, possibly due to the smaller pore radius.

### 4.2. Nanofiltration of Alcohol-Based Molecules

**Figure 2:**
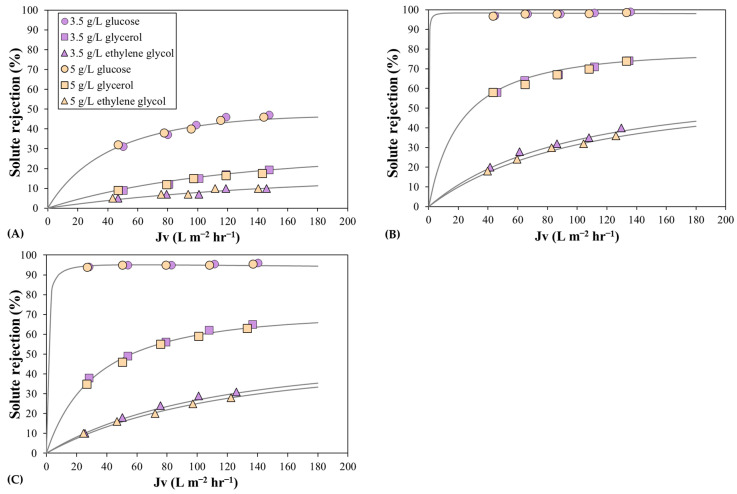
Rejection of glucose, glycerol, and ethylene glycol solutions as a function of the permeate flux: (**A**) the MPF-36 membrane; (**B**) the MPF-34 membrane; and (**C**) the Desal 5DK membrane. Symbols correspond to the experimental data, and lines correspond to the fitted data. Circles represent the feed concentration of 3.5 g L^−1^ and triangles 5 g L^−1^.

The NF membrane performance was characterized by the rejection of alcohol-based molecules and the water permeability coefficient. Figure 2 shows the observed rejections of glucose, glycerol, and ethylene glycol as a function of permeate flux for the three NF membranes at two feed solute concentrations. The membrane with the highest MWCO showed the lowest rejection values for the three solutes. The glucose molecule, which had the highest Stokes radius among the three solutes, showed the highest rejection for the three membranes, especially the MPF-34 and DK membranes, where the rejection was close to unity. Moreover, the MPF-34 membrane showed the highest rejection values, as expected from the water permeability coefficients. The lowest rejections values corresponded to ethylene glycol, which has the lowest molecular weight. Regarding the feed solute concentration effect, the observed glucose and glycerol rejections are almost independent of the solute concentration, and conversely, the observed ethylene glycol rejection decreases slightly with the solute concentration, especially for the MPF-34 and Desal 5DK membranes.

The experimentally observed rejections were fitted following the modeling procedure described in the Modeling subsection. Then, the hydrodynamic coefficient and the effective membrane thickness were estimated to match the experimental and calculated observed rejection with Equation (17) and using the corresponding mass transfer coefficient with the permeation correlation. Figure 2 also shows the theoretical curves that best fit the experimental data with the lower least-squares objective function $LS_{R,s}$. In general, there is a good agreement between the calculated and experimental rejections.

For the MPF-36 membrane, the experimentally observed rejections of the three solutes were fitted by one hydrodynamic coefficient for the two solute concentrations. Nevertheless, it is important to note that the glycerol and ethylene glycol rejections, which are below 20%, could be represented by different hydrodynamic coefficients with good correlation. This fact means that very low rejections lead to inconsistent model parameters, and it is desirable to use solutes with high maximum observed rejection, for example, between 50% and 90%, as shown in Figure 2. The estimated values found in the predictions are shown in Table 2. The hydrodynamic coefficient decreased with the solute molecular weight, and the effective membrane thickness has almost the same value for glucose and glycerol, and it increased slightly for ethylene glycol. By considering the solute Stokes radii, the mean membrane pore radius may be calculated from the hydrodynamic coefficient ($r_{p}=r_{s}/\lambda_{s}$) and its value is different for the three solutes. This discrepancy could be due to the lower precision in the fitting procedure for low rejection values. Therefore, the mean membrane pore radius and effective membrane thickness for the MPF-36 membrane are 0.855 nm and 3.92 μm, respectively, with regard to glucose and glycerol rejections. These characteristic parameters have not been widely documented. Sabaté et al. [30] found a membrane pore radius of 0.86 nm and an effective membrane thickness of 6.51 μm. Such a high value may result from using another equation (Hagen–Poiseuille equation) to evaluate the effective membrane thickness, which was calculated directly from the pure water permeability coefficient, and the concentration polarization phenomenon was not considered. Furthermore, the experimental work was performed at a temperature of 25 °C, which is higher than the temperature used in our work.

The rejection tendency shown for the MPF-34 and Desal 5DK membranes is very similar, and slightly higher rejection values are observed for the MPF-34 membrane, which is also mentioned in the literature [31]. Very high experimental values of glucose rejection led to inconsistent simulations, and different values of model parameters can simulate experimental data with good agreement. For instance, glucose rejection can be fitted with a hydrodynamic coefficient between 0.875 and 0.915 for the MPF-34 membrane and between 0.795 and 0.825 for the Desal 5DK membrane. Therefore, for a more accurate determination of the model parameters, the hydrodynamic coefficient and effective membrane thickness are usually calculated as the average of the estimated values from the experimental rejections of various uncharged solutes, such as glycerol, ethylene glycol, or ethanol [32,33]. In this context, glycerol rejection was almost independent of the feed concentration, and was 60 and 70% at 144 L m^−2^ hr^−1^ for MPF-34 and Desal 5DK membranes. Glycerol rejection was fitted using the same values of model parameters for the two feed concentrations and the least-squares objective function was lower than 0.6. To validate the fitted model parameters, a slight change was made in the hydrodynamic coefficient and the least-squares objective function increased to values above 10%, thus obtaining a worse correlation between the calculated and experimental rejection. By considering the solute Stokes radius for glucose and glycerol molecules, the mean membrane pore radius is 0.408 and 0.453 nm, and the mean effective membrane thickness is 1.95 and 2.28 μm for the MPF-34 and Desal 5DK membranes, respectively. These calculated values are consistent with values reported in the literature, although most of these were tested at 25 °C [30,32,34].

In contrast, the ethylene glycol rejections for the two low MWCO membranes showed a weak dependence on the solute concentration, resulting in different values for the model parameters. Subsequently, the solute rejection decreased as the solute concentration increased, and the hydrodynamic coefficient also fell. The membrane pore radius calculated with the Stokes radius changed from the values calculated with glucose and glycerol rejection and was 0.416 and 0.464 nm, respectively, for 3.5 g/L and for MPF-34 and Desal 5DK membranes. In addition, an increase in the effective membrane thickness was observed. These changes in the characteristic parameters of the model indicate a different membrane performance with the ethylene glycol molecule. It is known that organic solvents, such as low-molecular-weight alcohols, modify the permeation performance of NF membranes via the differences between their physical characteristics and those of water [35]. The presence of relatively low concentrations of alcohol molecules alters solution viscosity, solvation of organic molecules, and swelling of the pore walls, affecting the pore radius of the membrane [36]. Thus, the limiting rejection of alcohol molecules depends on their concentration in the solution, with the estimated pore radius varying significantly due to changes in the solvent–membrane interactions [37].

**Figure 3:**
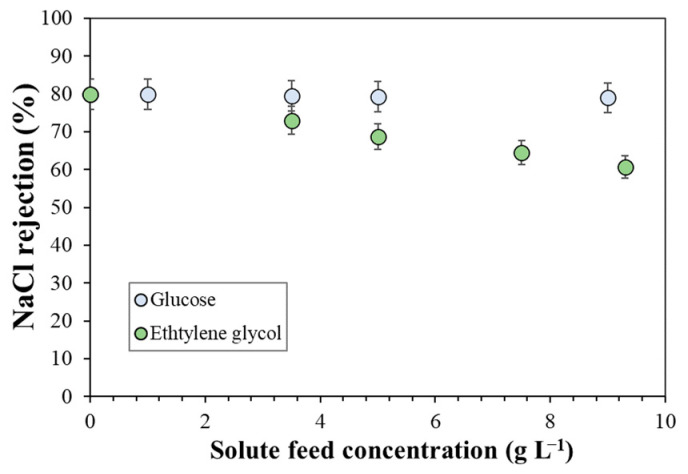
NaCl rejection as a function of glucose and ethylene glycol feed concentrations at constant permeate flux of 144 L m^−2^ hr^−1^ for the Desal 5DK membrane. NaCl feed concentration was 0.5 g L^−1^.

To assess membrane behavior in the presence of organic alcohol solutes, NaCl rejection was measured at different concentrations of glucose and ethylene glycol in water solutions, with a fixed NaCl feed concentration of 0.5 g L^−1^. Figure 3 shows the experimental data measured at a constant permeate flux of 144 L m^−2^ hr^−1^ for the Desal 5DK membrane. It should be noted that NaCl rejection was independent of the feed glucose concentration but decreased with increasing in the feed concentration of ethylene glycol. Therefore, these results showed that the alcohol-based molecules could alter the permeation process and the nanofiltration performance, with the estimation of the pore radius depending on the type and concentration of the alcohol solute.

**Figure 4:**
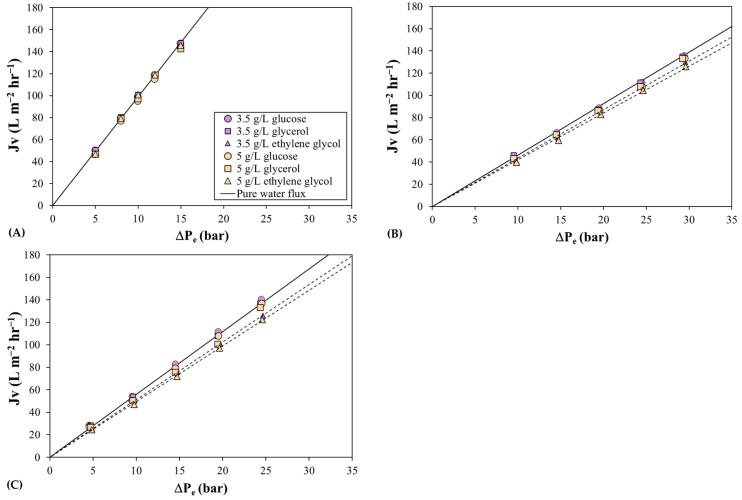
Permeate flux as a function of effective transmembrane pressure for glucose, glycerol, and ethylene glycol solutions: (**A**) the MPF-36 membrane; (**B**) the MPF-34 membrane; and (**C**) the Desal 5DK membrane. Symbols correspond to the experimental data and solid lines correspond to pure water flux and dashed lines correspond to the fitted data for ethylene glycol solutions. Circles represent the feed concentration of 3.5 g L^−1^ and triangles 5 g L^−1^.

The change in membrane performance in the presence of ethylene glycol molecules was also observed in the permeability coefficient. Figure 4 shows the permeate flux measured as a function of the effective transmembrane pressure, which was calculated considering the ideal solution using the Van’t Hoff equation and the corresponding solute mass transfer coefficients for the three membranes. As observed by other researchers [37,38], the permeability coefficient in the presence of alcohol-based molecules is lower than that obtained with pure water. For instance, the water permeability coefficient was reduced by 10% for a 5 g/L solution of ethylene glycol for the Desal 5DK membrane. This reduction in the permeability may be due to the different affinity between water and ethylene glycol molecules for hydrophilic membranes, which may increase the resistance to the solvent flux at the solution–membrane interface [37]. Nevertheless, changes in permeation features were not observed with the MPF-36 membrane (Figure 4), which could be due to low ethylene glycol rejection values.

### 4.3. Nanofiltration of Amino Acids

The rejections of triglycine, which is a small peptide composed of three glycine molecules, and glycine, which is a relatively small amino acid, were analyzed at pH 6. Based on the pK_a_ values of the two molecules, the net charge of triglycine and glycine is lower than 0.3% [39]. In the theoretical simulations, electrostatic charge effects, such as the screening of the Donnan effect and dielectric exclusion, can be avoided, and the main exclusion mechanism is the steric effect alone. In previous simulations, the calculated triglycine and glycine rejections showed no significant differences with changes in the effective membrane charge density and pore dielectric constant. The differences were found to be less than 2%, supporting the hypothesis that triglycine and glycine behave as zero-charged molecules at the pH tested. Therefore, the experimental rejections were fitted with the SPM described in the Theory section.

**Figure 5:**
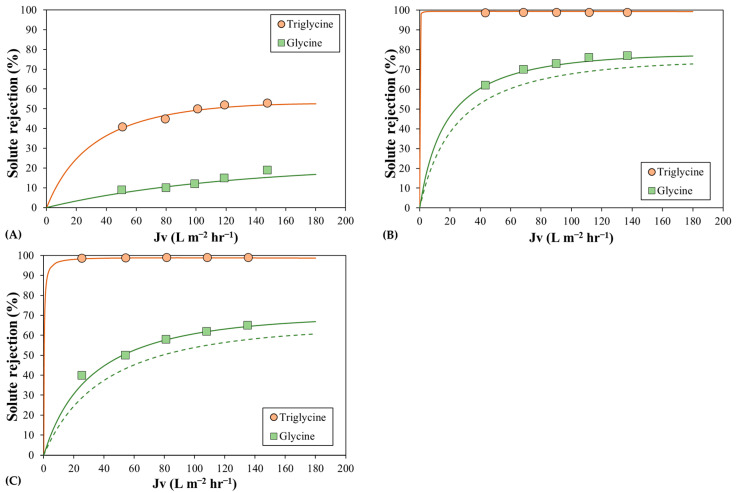
Rejection of triglycine and glycine as a function of permeate flux for (**A**) the MPF-36 membrane; (**B**) the MPF-34 membrane; and (**C**) the Desal 5DK membrane. Symbols correspond to experimental data, solid lines correspond to the best-fit curve, and dashed lines correspond to the calculated curve with glucose and glycerol model parameters.

Figure 5 shows the experimental and calculated rejection values of triglycine and glycine for the three membranes. The values of the model parameters are shown in Table 2. The triglycine rejection in Figure 5 showed remarkably high values for the MPF-34 and Desal 5DK membranes, invalidating the determination of the two model parameters. Thus, the observed rejection value was greater than 99%, which means that the mean pore size of both membranes is close to the triglycine radius. A similar effect has been observed for the glucose molecule and other larger molecules [40]. However, the limiting rejection of triglycine in the MPF-36 membrane was about 50%, and the best agreement between the model data and the experimental data was obtained with a hydrodynamic coefficient of 0.451 and an effective membrane pore thickness of 4.12 μm. Based on the solute Stokes radius, the estimated membrane pore radius for the MPF-36 membrane is 0.399 nm, which is similar to the estimated radius for glucose and glycerol molecules. This similar behavior of these three molecules is also observed in the solvent permeate flux versus effective transmembrane pressure (Figure 4).

The rejection of glycine is also shown in Figure 5. Its rejection values in the MPF-36 membrane were so low that the values of the model parameters could not be discriminated. MPF-34 and Desal 5DK membranes showed limiting glycine rejection of 80% and 70%, respectively, and the model parameters could be estimated accordingly [41]. First, the curves were calculated using the mean hydrodynamic coefficient and the mean effective membrane thickness, which were estimated from the uncharged solute rejections, but they did not fit well with the experimental data and underestimated the rejection values, as shown in Figure 5. Therefore, the best agreement between the calculated curves and the experimental data, which was found by minimizing the least-squares function between them, was achieved with the hydrodynamic coefficient of 0.634 and the effective membrane thickness of 2.09 μm for the MPF-34 membrane and 0.580 and 2.20 μm for the Desal 5DK membranes. These effective membrane thickness results are consistent with those of other authors when the concentration polarization was included in the model [42,43,44]. The membrane pore radius was estimated from the solute Stokes radius and was 0.386 nm and 0.422 nm for MPF-34 and Desal 5DK membranes, respectively, values slightly lower than those calculated with alcohol-based rejections. In the literature, there are different values for the membrane pore radius for the Desal 5DK membrane, ranging between 0.433 and 0.45 nm [32,34,42,43]. The model that did not considers the concentration polarization phenomenon estimated the membrane pore radius to be around 0.45 nm, while the models with concentration polarization showed lower values, similar to our study. In this regard, Bargeman et al. [43] determined the membrane pore radius and the effective membrane thickness of 0.42 nm and 2.59 μm, considering the concentration polarization and the Maxwell–Stefan equation for the solute transport inside the membrane. Consequently, it can be concluded that the rejection of small amino acid molecules can predict the performance of NF membranes with low MWCO at pH between 5 and 6, when a zero-charged molecule can be considered.

**Figure 6:**
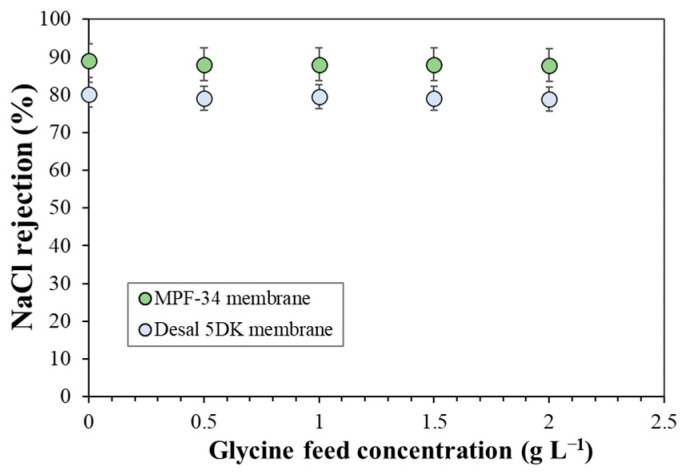
NaCl rejection as a function of glycine feed concentrations at constant permeate flux of 144 L m^−2^ hr^−1^ for MPF-34 and Desal 5DK membranes. NaCl feed concentration was 0.5 g L^−1^.

Regarding the permeate flux (Figure 4), the solvent permeability coefficient calculated for the triglycine and glycine solutions was very similar to the coefficients calculated for the glucose solutions for the three membranes, meaning that amino acids and glucose do not affect the membrane material and that the solvent permeation is similar to that of pure water. This argument is strengthened by the evolution of NaCl rejection as a function of glycine concentration (Figure 6), which remains nearly constant for both membranes. Hence, the performance of the NF membranes of low MWCO can be assessed better by nanofiltration of amino acid solutions than by a solution of alcohol-based molecules.

Based on the results of the experiments presented in this work, it appears that the Desal 5DK membrane may be able to partially separate glucose or triglycine and glycine in binary solutions. Monosaccharides and tripeptides show rejection values close to unity, and the amino acid rejection was less than 65% at permeate fluxes up to 144 L m^−2^ hr^−1^. The separation process can be carried out in continuous diafiltration mode, which consists of continuously adding water to maintain the feed volume constant [45]; this keeps the concentration of the large solute constant in the feed tank and prevents fouling of the membrane [46,47]. Therefore, future work will be based on experimental studies on the separation of monosaccharides and amino acids in continuous diafiltration mode using nanofiltration membranes.

## 5. Conclusions

The rejection of organic solutes and salt solutions in three different nanofiltration membranes was assessed with the SPM model at pH 6. A high level of concordance between the calculations and the experimental data. The use of uncharged organic solutes or zero net charge molecules, such as amino acids and small peptides, has been confirmed to be a good strategy for determining the mean membrane pore radius, especially for NF membranes with a low molecular weight cut-off, at pH values between 4.5 to 6.5. The membrane pore radius of these membranes is usually estimated from the rejection values of organic solutions, which are alcohol-based molecules. The performance of the membrane was found to be different in the presence of relatively low concentrations of ethylene glycol. Therefore, these low molecular weight alcohol molecules can interact with the membrane matrix and change its physical properties, leading to an overestimation of the membrane pore radius.

## Figures and Tables

**Table 1:** Physical properties of the solutes used in this study.

Species	MW (g mol^−1^)	pka_1_	pka_2_	r_s_ ^b^ (nm)	D_s_ (10^9^ m^2^ s^−1^)
Glucose	180.2	-	-	0.365	0.586
Glycerol	92.1	-	-	0.260	0.950
Ethylene glycol	62.07	-	-	0.211	1.014
Triglycine	189.2	3.23	8.09	0.375 ^a^	0.571
Glycine	75.0	2.37	9.60	0.245	0.873

^a^ rs calculated using the chemistry software ChemSketch, Version 11 (ACD/Labs, Inc., Toronto, ON, Canada) by means of atomic additive increments. ^b^ rs corresponding to the Stoke’s radius.

**Table 2:** Model parameters obtained by fitting the experimental data.

	MPF-36 Membrane					
Solute	λ (-)	δ (μm)	rp ^b^ (nm)	LS_R,s_	Lp (L m^−2^ hr^−1^ bar^−1^)	LS_Jv,s_
Glucose	0.430	3.96	0.848	0.696	9.97	0.162
Glycerol	0.302	3.88	0.961	0.256	10.1	0.233
Ethylene glycol	0.237	4.68	0.890	0.520	10.4	0.364
Glycine	0.285	3.86	0.859	0.932	10.1	0.321
Triglycine	0.451	4.12	0.832	0.311	10.0	0.248
	**MPF-34 membrane**					
	λ (-)	δ (μm)	rp ^b^ (nm)	LS_R,s_	Lp (L m^−2^ hr^−1^ bar^−1^)	LS_Jv,s_
Glucose	0.892	2.01	0.409	0.135	4.57	0.138
Glycerol	0.636	1.88	0.409	0.206	4.61	0.214
Ethylene glycol ^a^	0.512–0.501	1.49–1.48	0.412–0.421	0.366–0.308	4.43–4.25	0.219–0.283
Glycine	0.634	2.09	0.386	0.211	4.61	0.206
Triglycine	0.938	1.92	0.399	0.0891	4.58	0.200
	**Desal 5DK membrane**					
	λ (-)	δ (μm)	rp ^b^ (nm)	LS_R,s_	Lp (L m^−2^ hr^−1^ bar^−1^)	LS_Jv,s_
Glucose	0.807	2.14	0.452	0.250	5.69	0.215
Glycerol	0.573	2.43	0.453	0.570	5.47	0.261
Ethylene glycol ^a^	0.455–0.449	1.97–1.91	0.463–0.470	0.393–0.489	5.22–5.08	0.108–0.153
Glycine	0.580	2.20	0.422	0.705	5.62	0.120
Triglycine	0.904	2.12	0.415	0.0435	5.47	0.128

^a^ The first value corresponds to 3.5 g/L and the second to 5 g/L ethylene glycol solutions. ^b^ The membrane pore radius (rp) was calculated from the solute Stoke radius.

## Data Availability

Not applicable.
